# Induction of apoptosis in prostate cancer by ginsenoside Rh2

**DOI:** 10.18632/oncotarget.24326

**Published:** 2018-01-27

**Authors:** Tony Tong-Lin Wu, Yat-Ching Tong, I-Hung Chen, Ho-Shan Niu, Yingxiao Li, Juei-Tang Cheng

**Affiliations:** ^1^ Institute of Medical Sciences, Chang Jung Christian University, Tainan, Taiwan; ^2^ Division of Urology, Department of Surgery, Kaohsiung Veterans General Hospital, Kaohsiung, Taiwan; ^3^ Department of Urology, School of Medicine, National Yang Ming University, Taipei, Taiwan; ^4^ Department of Urology, National Cheng Kung University Hospital, College of Medicine, National Cheng Kung University, Tainan, Taiwan; ^5^ Department of Nursing, Tzu Chi University of Science and Technology, Hualien, Taiwan; ^6^ Department of Medical Research, Chi-Mei Medical Center, Tainan, Taiwan

**Keywords:** prostate cancer, apoptosis, ginsenoside Rh2, peroxisome proliferator-activated receptor

## Abstract

The therapeutic action of ginsenoside Rh2 on several cancer models has been reported. This study aimed to evaluate its apoptotic effect on prostate cancer and the underlying mechanism. Cultured DU145 cells were treated with Rh2 (5 × 10^–5^ to 1 × 10^–4^ M), peroxisome proliferator-activated receptor-delta (PPAR-delta) antagonist GSK0660 (1 × 10^–6^ to 5 × 10^–6^ M); or small interfering RNA (siRNA) of PPAR-delta. The treatment effects were evaluated with cell viability assay, life/death staining and flow cytometry for apoptosis. Immunostaining was used for reactive oxygen species (ROS) and superoxide detection. Western blot analysis for PPAR-delta and signal transducer and activator of transcription 3 (STAT3) protein expression were performed. The results showed that Rh2 significantly decreased DU145 cell survival and increased cell apoptosis. ROS and superoxide induction, PPAR-delta up-regulation and phosphorylated STAT3 (p-STAT3) down-regulation by Rh2 were demonstrated. GSK0660 partially but significantly inhibited the Rh2-induced apoptosis and restored cell viability. Treatment with siRNA reversed the Rh2-induced apoptosis as well as changes in PPAR-delta and p-STAT3 expression. In conclusion, our findings have demonstrated that ginsenoside Rh2 induces prostate cancer DU145 cells apoptosis through up-regulation of PPAR-delta expression which is associated with p-STAT3 up-regulation and ROS/superoxide induction. Rh2 may be potentially useful in the treatment of prostate cancer.

## INTRODUCTION

Prostate cancer is one of the most common malignancies in men worldwide [1]. When the cancer is localized within the prostate gland, it can usually be treated with surgery or radiotherapy with good prognosis. However, the mortality rate increases significantly when the cancer cells metastasize beyond the gland. Androgen deprivation therapy with medical or surgical castration is the mainstay treatment for metastatic prostate. However, the treatment becomes ineffective when castration-resistant prostate cancer (CRPC) eventually developed. Currently effective treatment options for CRCP are quite limited and development of new therapeutic agents will greatly benefit the CRPC patients [2].

Ginsenosides are a class of natural components extracted from the plant ginseng which has been used in traditional medicine for long time. The ginsenosides have also been known for their medicinal effects such as anti-inflammatory and anti-proliferative activities [3, 4]. Ginsenoside Rh2 was isolated and identified as an anti-tumor constituent by Chen *et al.* [5]. Thereafter, its anti-cancer activities have been reported in various malignant diseases including ovarian cancer, breast cancer and melanoma [6–8]. It was demonstrated that Rh2 could induce cell apoptosis through activation of caspase-3 protease [9]. For prostatic cancer, Rh2 inhibited proliferation of androgen-dependent and -independent prostate cancer cells [10]. It has also been shown that Rh2 could inhibit growth of prostatic cancer both *in vivo* and *in vitro*. Moreover, the treatment effect was due to a combined inhibitory action on tumor cell proliferation and invasiveness [11].

Peroxisome proliferator-activated receptors (PPARs) are a group of nuclear receptors that function as transcription factors for the regulation of gene expression. There are three PPAR isoforms: PPAR-gamma, PPAR-alpha and PPAR-delta (also named as PPAR-beta and PPAR-beta/delta) [12] The PPARs are involved in a wide range of cellular functions including proliferation, differentiation, development and metabolism. Pathologically, the receptors are related to atherosclerosis, inflammation, cancer, infertility, and degenerative diseases. Recent evidence has shown the relationship between PPAR-delta expression and apoptosis in prostate cancer DU145 cells [13]. The DU145 cell line was derived from human prostate adenocarcinoma metastatic to the brain [14]. The cells are hormone-insensitive and thus a useful model for studying CRPC. In the present study, the apoptotic effect of Rh2 on DU145 cells and the possible underlying mechanism(s) involving PPAR-delta were investigated.

## RESULTS

### Effect of Rh2 on DU145 cell survival

**Figure 1:**
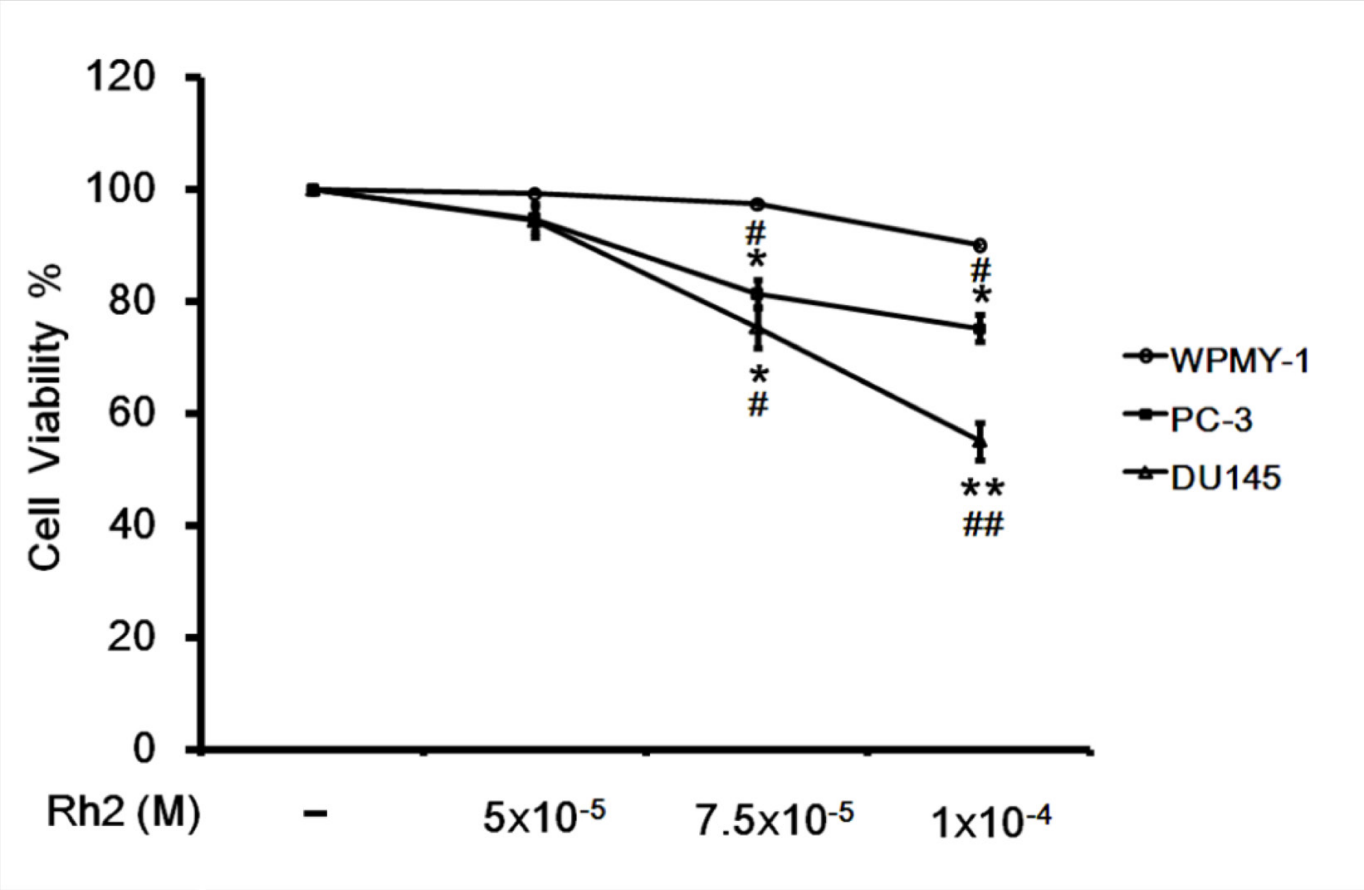
Cell viability assay showing the inhibitory effect of Rh2 on DU145 cell viability DU145 cells were incubated with Rh2 (5 × 10^–5^ to 1 × 10^–4^ M) for 24 h. Human prostate cancer PC-3 cells and prostate stromal myofibroblast cell WPMY-1 were used as controls. Rh2 significantly reduced cell viability of DU145 and PC-3 cells in a concentration-dependent manner but not the WPMY-1 cell. The data are expressed as the means ± S.E.M. (*n =* 8 for each group). ^*^P < 0.05 and ^**^P < 0.01 compared with control DU145 cells; ^#^*P* < 0.05 and ^##^*P* < 0.01 compared with WPMY-1 cells treated with Rh2.

**Figure 2:**
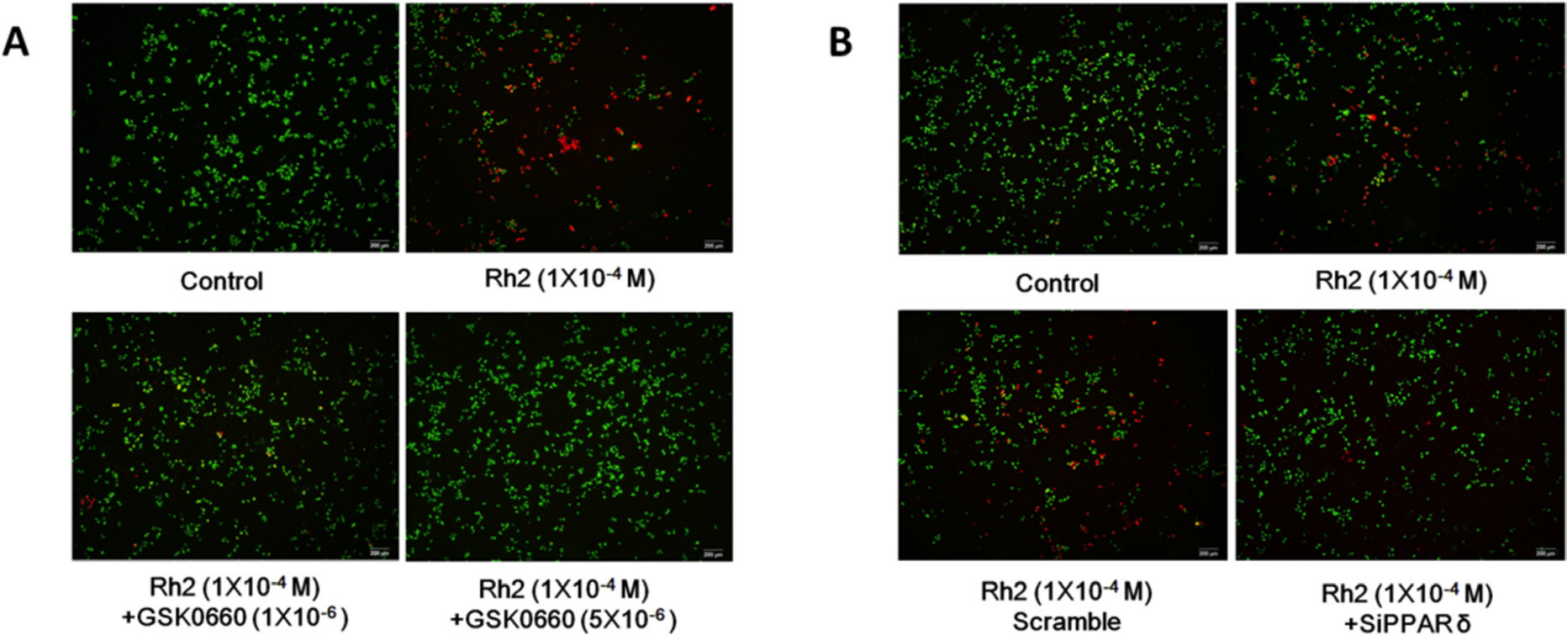
Live/dead cell staining showing GSK0660 and siRNA inhibition on Rh2 apoptotic effect DU145 cells were incubated with Rh2 (1 × 10^–4^ M) with/without GSK0060 (1–5 × 10^–6^ M) for 24 h or transfected with PPAR-delta siRNA 48 h prior to Rh2 treatment. Live cells were stained green, whereas dead cells are stained red. (**A**) Co-incubation with GSK0660 (1–5 × 10^–6^ M) inhibited the Rh2 apoptotic effect. (**B**) PPAR-delta siRNA (SiPPARδ) but not scramble siRNA (Scramble) inhibited the Rh2 apoptotic effect on DU145 cells.

**Figure 3:**
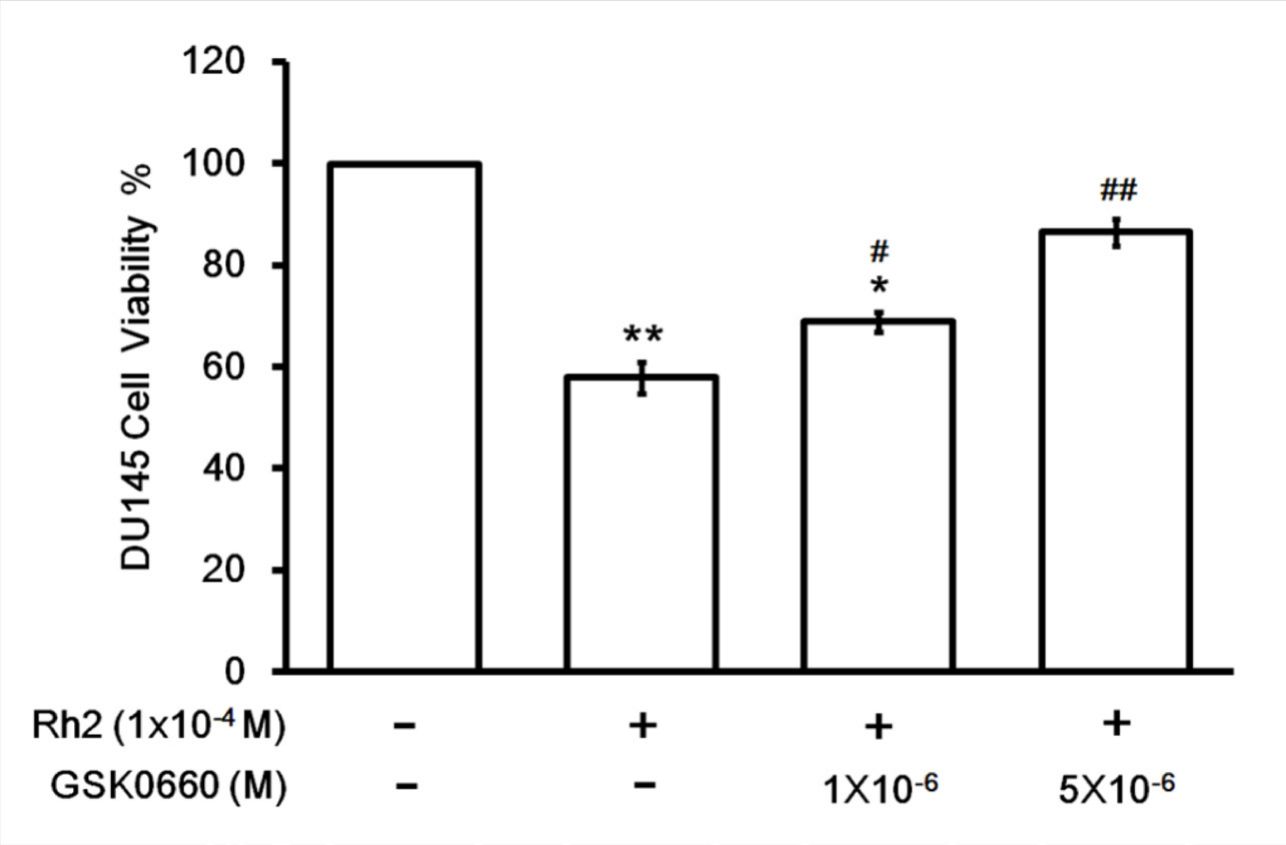
Cell viability assay showing GSK0660 inhibition on Rh2 apoptotic effect DU145 cells were incubated with Rh2 (1 × 10^–4^ M) with/without GSK0060 (1–5 × 10^–6^ M) for 24 hours. The bars depict the quantitative data of cell viability assay on DU145 cells. The data are expressed as the means ± S.E.M. (*n =* 8 for each group). ^*^P < 0.05 and ^**^P < 0.01 compared with control DU145 cells; ^#^*P* < 0.05 and ^##^*P* < 0.01 compared with DU145 cells treated with Rh2 only.

**Figure 4:**
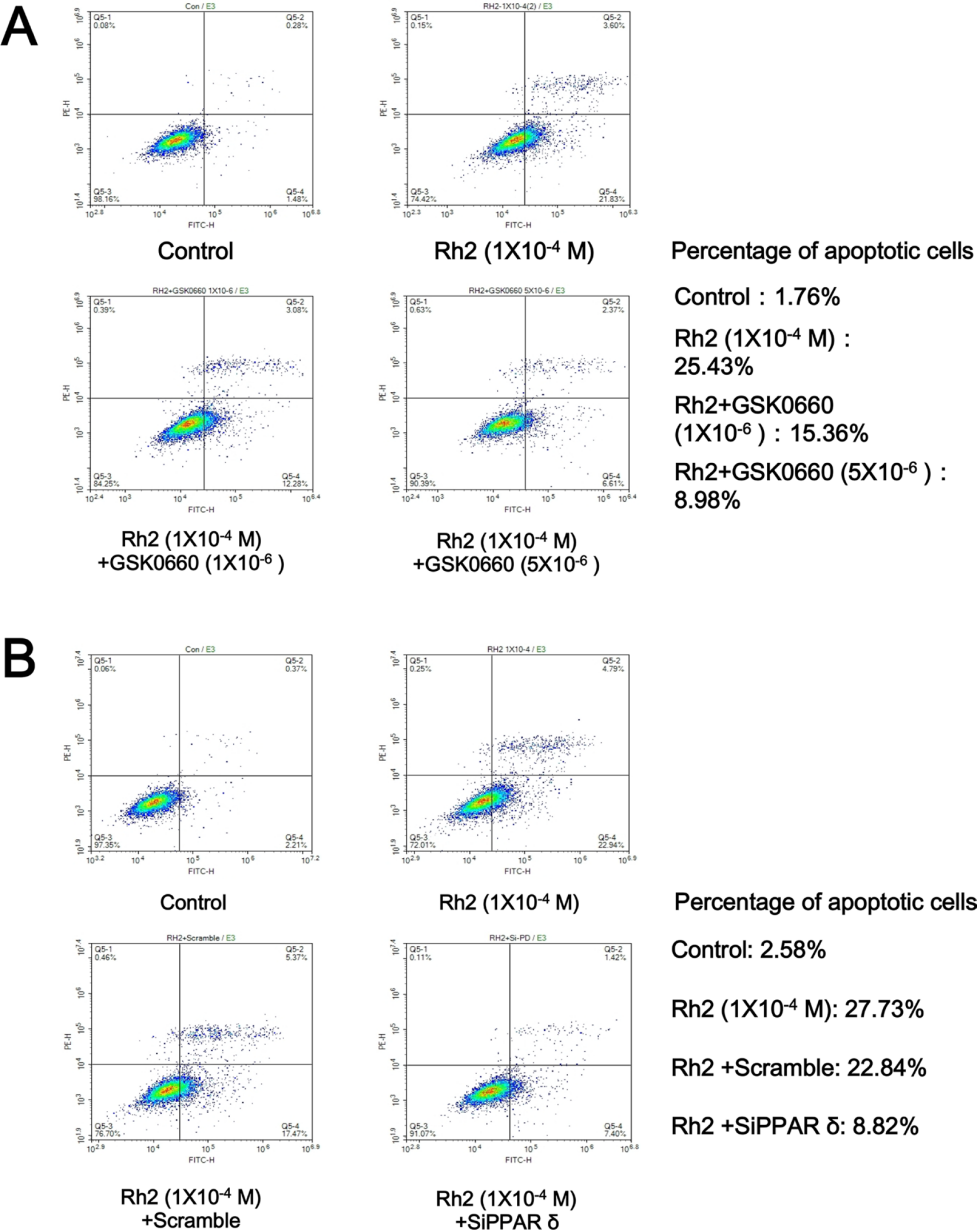
Flow cytometry showing GSK0660 and siRNA inhibition on Rh2 apoptotic effect DU145 cells were incubated with Rh2 (1 × 10^–4^ M) with/without GSK0060 (1–5 × 10^–6^ M) for 24 h or transfected with PPAR-delta siRNA 48 h prior to Rh2 treatment. or PPAR-delta siRNA for 24 hours. (**A**) Rh2 significantly increased the percentage of apoptotic cells; the effect was inhibited by GSK0660. (**B**) Rh2 significantly increased the percentage of apoptotic cells; the effect was inhibited by PPAR-delta siRNA (SiPPARδ) but not scramble siRNA (Scramble).

Rh2 incubation in culture medium reduced DU145 cell viability significantly in a dose-dependent fashion (Figures 1, 2). At a concentration of 1 × 10^–4^ M, the percentage of viable DU145 cells fell to about 50% (Figure 3). On the other hand, flow cytometry showed that Rh2 induced significant increase in the percentage of apoptotic DU145 cells (Figure 4). Co-incubation with GSK0660 or pre-treatment with PPAR-delta small interfering RNA (siRNA) could reverse these changes.

### Effect of Rh2 on PPAR-delta, p-STAT/STAT3 protein expression

**Figure 5:**
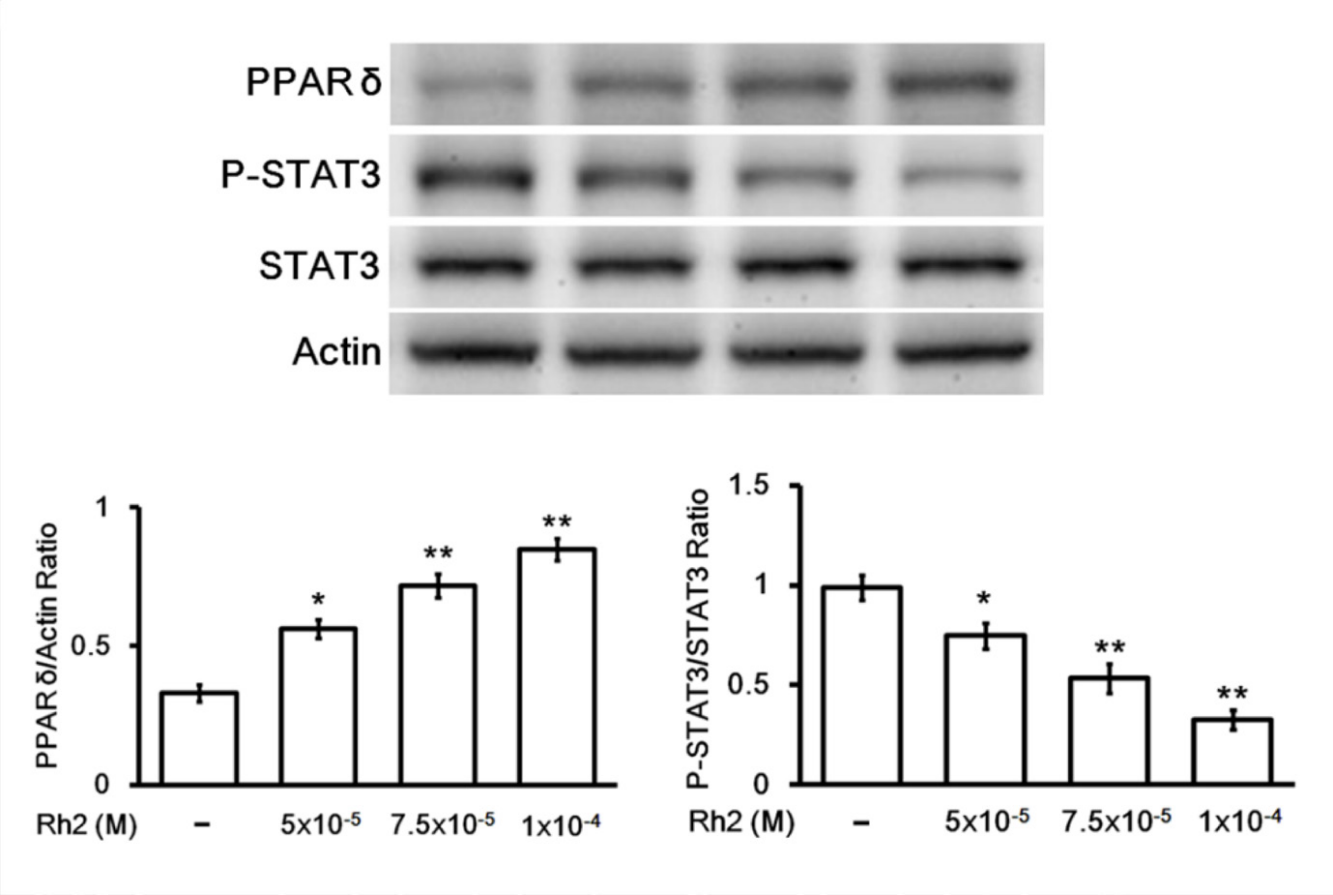
Western blot showing Rh2 effects on PPAR-delta and p-STAT3/STAT3 protein expression DU145 cells were incubated with Rh2 (5 × 10^–5^ to 1 × 10^–4^ M) for 24 h. Rh2 significantly increased PPAR-delta and decreased P-STAT3/STAT3 expression in a concentration-dependent manner. The data are expressed as the means ± S.E.M. (*n =* 8 for each group). ^*^P < 0.05 and ^**^P < 0.01 compared with control DU145 cells.

**Figure 6:**
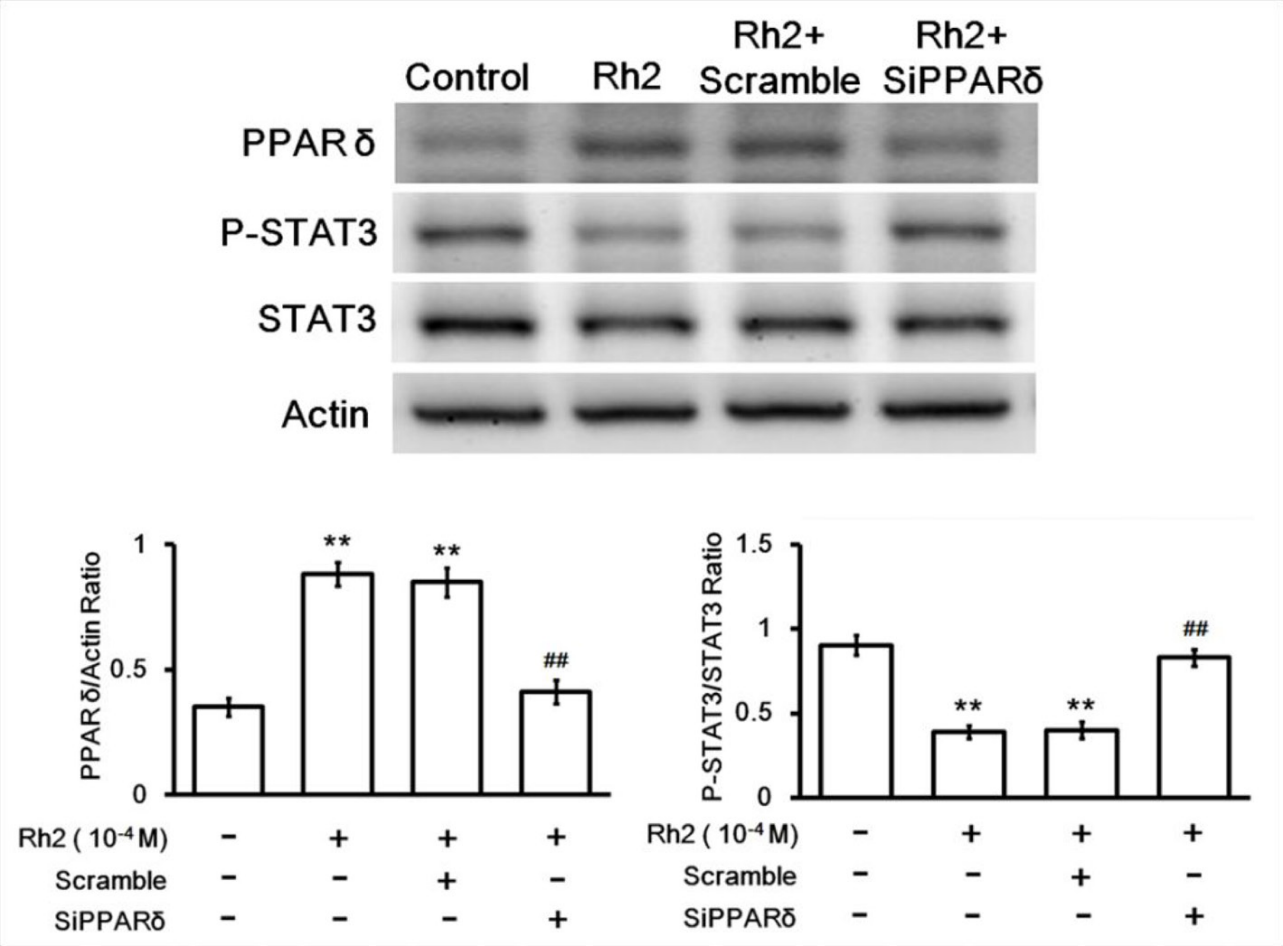
Western blot showing siRNA inhibition on Rh2-induced PPAR-delta and p-STAT3/STAT3 changes DU145 cells were transfected with PPAR-delta siRNA 48 h prior to incubation with Rh2 (1 × 10^–4^ M). Rh2 significantly increased PPAR-delta and decreased P-STAT3/STAT3 expression. PPAR-delta siRNA (SiPPARδ) but not scramble siRNA (Scramble) inhibited the Rh2-induced changes. The data are expressed as the means ± S.E.M. (*n =* 8 for each group). ^**^P < 0.01 compared with control DU145 cells; ^##^*P* < 0.01 compared with DU145 cells treated with Rh2 only.

Western blots showed that Rh2 significantly increased DU 145 cell PPAR-delta protein expression. On the contrary the activated form of signal transducer and activator of transcription 3 (STAT3), phosphorylated-STAT3 (p-STAT3), was significantly decreased (Figure 5). Treatment with PPAR-delta siRNA inhibited these changes (Figure 6).

### Rh2 induction of intracellular ROS and superoxide

**Figure 7:**
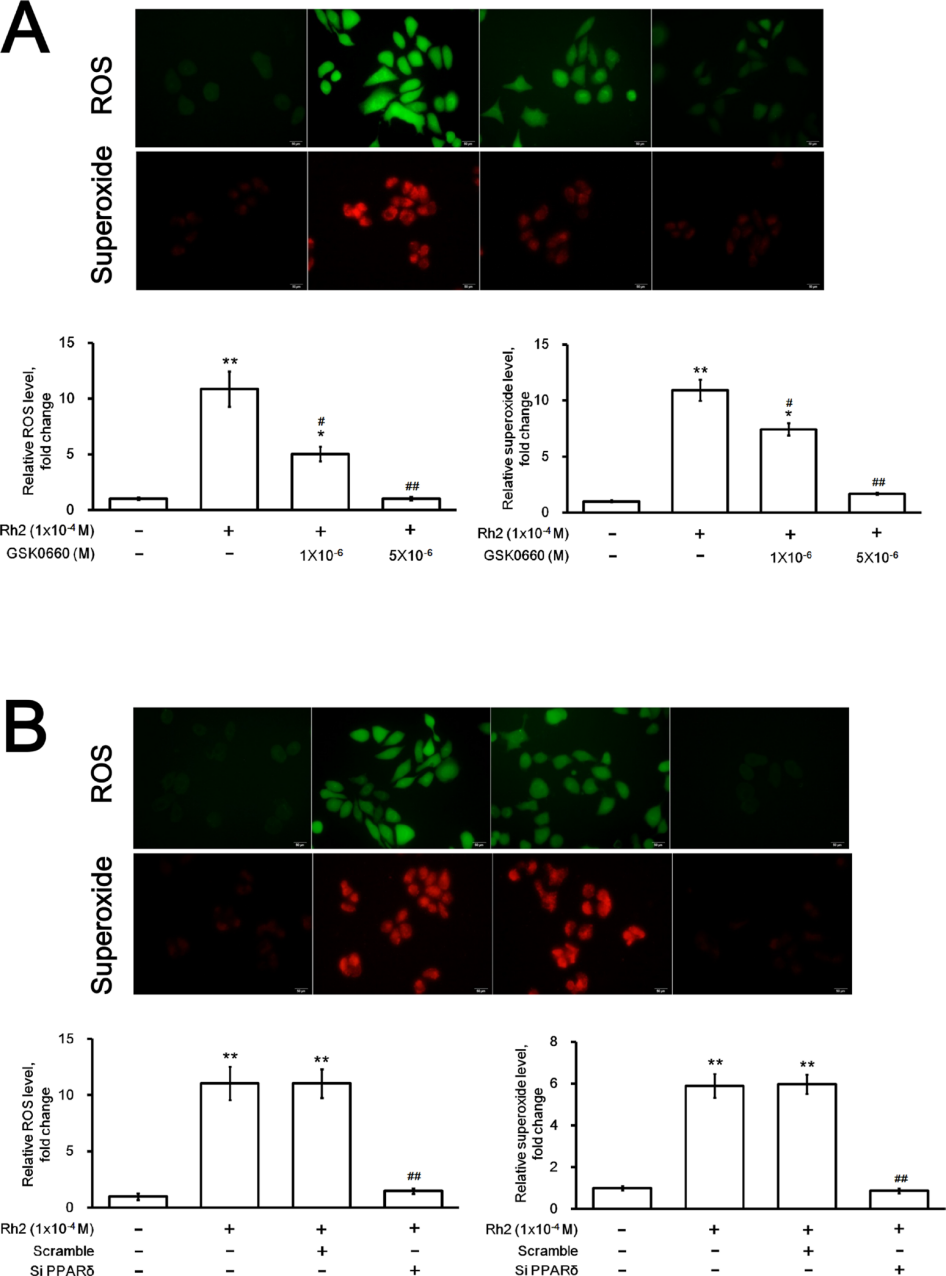
Fluorescence microscopy showing intracellular ROS and superoxide formation DU145 cells were incubated with Rh2 (1 × 10^–4^ M) with/without GSK0060 (1–5 × 10^–6^ M) for 24 h or transfected with PPAR-delta siRNA 48 h prior to Rh2 treatment. or PPAR-delta siRNA for 24 hours. (**A**) Rh2 significantly increased intracellular ROS (green) and superoxide (red); the effect was inhibited by GSK0660. (**B**) Rh2 significantly increased intracellular ROS and superoxide; the effect was inhibited by PPAR-delta siRNA (SiPPARδ) but not scramble siRNA (Scramble). The data are expressed as the means ± S.E.M. (*n =* 8 for each group). The ROS and superoxide level in the control DU145 cells are taken as 1 arbitrary unit. ^*^P < 0.05 and ^**^P < 0.01 compared with control DU145 cells; ^#^*P* < 0.05 and ^##^*P* < 0.01compared with DU145 cells treated with Rh2 only.

Figure 7 showed that DU145 intracellular ROS and superoxide were significantly increased after incubation with Rh2. Treatment with GSK0660 (Figure 7A) or PPAR-delta siRNA (Figure 7B) could both reverse these effects.

## DISCUSSION

Our study has demonstrated that ginsensodie Rh2 could effectively control prostate cancer growth by its apoptotic effect on cancer cells. Both DU145 and PC-3 cell viability were significantly reduced by Rh2 in our experiment. PC-3 cell line was originally established from human grade-4 prostate cancer metastatic to the brain and has a greater metastatic potential than DU145 cells [15]. On the contrary, Rh2 did not exhibit apoptotic effect towards the benign control WPMY-1 cells which was cultured from prostate stromal myofibroblast. Therefore Rh2 may be potentially useful in the treatment of metastatic or hormonal refractory prostatic cancer.

The possible role of PPAR-delta in prostate cancer cells apoptosis was reported recently. Similar to our current finding, it has been shown the telmisartan, an angiotensin II receptor blocker, induced DU145 cell apoptosis through PPAR-delta up-regulation [13]. Previous evidence has demonstrated the relationship between PPARs and various kinds of malignant diseases [16–18]. PPAR-gamma activation in cultured breast cancer cells induced lipid accumulation and gene expression changes associated with a more differentiated, less malignant state [19]. It was therefore postulated that PPAR-gamma transcriptional pathway could induce differentiation of malignant breast epithelial cells and provide a potential therapy for breast cancer. As for the prostate, the expression of PPAR-gamma was found significantly higher in high-risk prostate cancer than in low-risk prostate cancer and benign prostatic hyperplasia [20]. In addition, PPAR agonists including troglitazone, rosiglitazone, ciglitazone and pioglitazone could inhibit proliferation of castration-sensitive (LNCaP) as well as castration-resistant (C4-2, PC-3 and DU145) prostate cancer cell lines [21–23]. Regarding PPAR-delta, it was reported that genetic knockdown of the receptor promotes colon cancer growth by decreasing differentiation, increasing proliferation and vascular endothelial growth factor expression in cancer cells [24]. The receptor could also promote breast cancer and leukemic cell survival cultured under unfavorable metabolic conditions [25, 26]. Contrary to these reported PPAR-delta oncogenic effects, studies have also shown possible tumor suppression actions by PPAR-delta. Inhibition of cell proliferation by PPAR-delta ligands were demonstrated in a number of tumors including lung cancer, colon cancer and neuroblastoma [27–29]. Therefore the role of PPAR-delta in cancer treatment is still controversial. It has been hypothesized that PPAR-delta may have tumor-promotion and tumor-suppression actions depending on the tissue, cell-type or organ in question [30].

Our results showed that p-STAT3/STAT3 down-regulation and ROS/ superoxide production are down-stream events associated with the Rh2-induced PPAR-delta up-regulation. STAT3 belongs to the STAT protein family which are intracellular transcription factors that mediate a variety of cellular functions including proliferation, apoptosis and differentiation. When stimulated by cytokines and growth factors, STAT3 is phosphorylated to p-STAT3 by the action of membrane-associated Janus kinases (JAK). The activated p-STAT3 is translocated into the cell nucleus and acts as a transcription activator that regulates gene expression. In the study of lung adenocarcinoma, it was shown that JAK/STAT3 signaling pathway participated in the apoptosis of PC-9 cells induced by icotinib. It has been postulated that icotinib inhibits the gene expression levels of JAK2 and STAT3 leading to up-regulation of the apoptosis regulator BAX gene [31]. The functional linkage between STAT3 and ROS has also been reported. In the A-431 carcinoma cell, platelet-derived growth factor activated STAT3 by using intracellular ROS as a second messenger [32]. Moreover, ROS regulated heat-shock protein 70 via the JAK/STAT pathway in vascular smooth muscle cells [33]. Research evidence demonstrates that ROS play an important role in malignant transformation through various cellular signaling pathways, STAT3 being one of the important ones [34]. On the other hand, it was shown that ROS is an important signaling intermediate leading to Rh2-induced apoptosis in HeLa, MCF10A-ras, and MCF7 cells [35]. In human leukemia Jurkat cells, Rh2 could induce mitochondrial-associated apoptosis by increasing mitochondrial ROS [36]. With relationship to the mitochondria-mediated apoptosis pathway, Rh2 exhibited the actions of release of mitochondrial cytochrome c, activation of caspase-3 and Bax protein, inhibition of Bcl-2 protein as well as production of intracellular reactive oxygen species in Hep3B cells. These findings suggest that Rh2 may induce apoptosis by direct activation of the mitochondrial pathway [37]. Taken together, our findings support previous studies of the inhibitory effect of Rh2 on prostate cancer growth [10, 11]. In addition, it is the first study showing the role of PPAR-delta in the Rh2-induced prostate cancer cell apoptosis. However, the exact underlying mechanism of this process is still unclear and will need further investigation.

In conclusion, our findings have shown that ginsenoside Rh2 induces prostate cancer DU145 cells apoptosis through up-regulation of PPAR-delta which leads to down-regulation of p-STAT3/STAT3 and intracellular oxidative stress. Rh2 may be potentially useful for the clinical treatment of prostate cancer.

## MATERIALS AND METHODS

### Culture of DU145 cells

DU145 cells (purchased from the Culture Collection and Research Center of the Food Industry Institute, Hsin-Chiu City, Taiwan) were incubated in α-MEM medium (Hyclone, Logan, UT, USA) with 10% heat-inactivated fetal bovine serum (FBS) (Gibco, Life Technologies, Kaohsiung, Taiwan). A humidified atmosphere of 95% air and 5% CO_2_ was kept at 37° C.

### Pharmacological study with Rh2 and GSK0660

When the cells reached 60% confluence, the culture was replaced with serum-free cell medium containing Rh2 (5 × 10^–5^ to 1 × 10^–4^ M; Tauto Biotech, Shanghai, China) with/without GSK0660 (a PPAR-delta antagonist, 1 × 10^–6^ to 5 × 10^–6^ M; Sigma-Aldrich, St. Louis, MO, USA). After drug incubation for 24 hours the cells were harvested by treatment with 0.25% trypsin and 0.2 g/L EDTA.

### PPAR-delta siRNA study

This method was previously reported [13]. In brief, duplexed RNA oligonucleotides for human PPAR-delta (siGENOME SMARTpool TM) were obtained from Thermo Fisher Scientific Inc., Taipei, Taiwan. DU145 cells were transfected with 40 pmol of PPAR-delta siRNA or scramble siRNA (scramble) using transfection reagents (TransIT-TKO, Mirus) as directed in the manufacturer’s protocols. The adequacy of PPAR-delta silencing was tested by immunoblot to optimize the experimental conditions (siRNA dose and time after transfection) according to previous reports [38–40].

### DU145 cell viability assay

The method employed for measuring DU145 cell viability by the 3- (4,5-cimethylthiazol-2-yl)-2,5-diphenyl tetrazolium bromide (MTT) assay was previously reported [41]. Briefly, 10^4^ cells were seeded on 96-well plates in 100 μl/well of fresh medium and 10 μl of MTT (final concentration of 0.5 mg/ml). The plates were incubated at 37° C for 4 h. During this time, viable cells were able to reduce the yellow tetrazolium salt into dark blue formazan crystals. The formazan crystals were dissolved using a solution of 0.01 M HCl/10% sodium docecyl sulfate (SDS). The absorbance of each well was by Synergy HT Multi-Mode Microplate Reader at 595 nm (BioTek, Winooski, VT, USA).

### Live/dead cell staining

Our method was previously reported [13]. A LIVE/DEAD viability assay kit (Molecular Probes; Eugene, OR, USA) was used according to the manufacturer’s instructions. DU145 cells were incubated with two probes, calcein-AM (green color) and ethidium homodimer-1 (EtdD-1, red color), for intracellular esterase activity and plasma membrane integrity respectively. The cells were then visualized under a fluorescence microscope (Olympus IX71; Olympus, Japan).

### Flow cytometry analysis

DU145 apoptotic cells measurement was performed by flow cytometry with Annexin V- propidium iodide staining [42]. FITC Apoptosis Detection Kit (BD Bioscience, San Diego, CA, USA) was used. DU145 cells were seeded in 12-well plates at a density of 5 × 10^5^ cells/well and the experiment procedure was performed according to the manufacturer’s protocol. Staining was analyzed by fluorescence-activated cell sorting (FACS).

### Western blot analysis

Our method was previously reported [13]. Extraction of protein from cell lysates was performed using ice-cold radio-immuno-precipitation assay (RIPA) buffer supplemented with phosphatase and protease inhibitors (50 mM sodium vanadate, 0.5 mM phenylmethylsulphonyl fluoride, 2 mg/mL aprotinin, and 0.5 mg/mL leupeptin). The protein concentrations were determined via the Bio-Rad protein assay (Bio-Rad Laboratories, Inc., Hercules, CA, USA). Total protein samples (30 μg) were separated via SDS/polyacrylamide gel electrophoresis (10% acrylamide gel) using the Bio-Rad Mini-Protein II system. The proteins were then transferred to polyvinylidene difluoride membranes (PerkinElmer, Waltham, MA, USA) using a Bio-Rad Trans-Blot system. The membrane was blocked with 5% non-fat milk in Tris-Buffered saline containing 0.1% Tween 20 (TBS-T). After incubation for two hours, the membrane was washed in TBS-T and hybridized with primary antibodies specific to PPAR-delta, STAT3 and p-STAT3 in TBS-T for 16 hours. b-actin was used as an internal control. Incubation with secondary antibodies and detection of the antigen–antibody complex were performed using an enhanced chemiluminescence (ECL) kit (Amersham Biosciences, Buckinghamshire, UK). The immunoblots of b-actin (43 kDa), PPAR-delta (55 kDa), STAT3/p-STAT3 (88 kDa) were quantified with a laser densitometer (Avegene Life Science, Taipei, Taiwan).

### Immunostaining for reactive oxygen species (ROS) and superoxide

For the detection of the intracellular ROS and superoxide, the Oxidative Stress Detection Kit from Thermo Fisher Scientific Inc. (Rockford, IL, USA) was used. The procedures were performed according to the manufacturer’s manual. The staining was visualized using a fluorescence microscope connected to an imaging system (IX71 Olympus, Tokyo, Japan).

### Statistical analysis

The data are presented as the mean ± S.E.M. for the number (n) of individual experiments. One-way analysis of variance (ANOVA) followed by a post hoc test was used to compare protein expression level changes and other parameters. Differences resulting in a *P*-value of 0.05 or less were considered to be statistically significant.

## Abbreviations

CRPC: castration-resistant prostate cancer
JAK: Janus kinases
PPAR: peroxisome proliferator-activated receptor
p-STAT3: phosphorylated signal transducer and activator of transcription 3
ROS: reactive oxygen species
STAT3: signal transducer and activator of transcription 3
